# Visual Discrimination Task in Guppies Using a Simultaneous Matching-to-Sample Procedure

**DOI:** 10.3390/ani15131936

**Published:** 2025-07-01

**Authors:** Gabriela Gjinaj, Marco Dadda, Maria Elena Miletto Petrazzini

**Affiliations:** 1 Department of General Psychology, University of Padova, Via Venezia 8, 35131 Padova, Italy; gabriela.gjinaj@unipd.it (G.G.); marco.dadda@unipd.it (M.D.); 2 Department of Biomedical Sciences, University of Padova, Via Ugo Bassi 58/B, 35131 Padova, Italy

**Keywords:** guppies, generalization, cognition, behavior

## Abstract

Cognitive abilities in fish have been widely demonstrated using experimental protocols commonly adopted with mammals and birds. Only a few studies have tested fish in the simultaneous match-to-sample task (sMTS), and mixed evidence regarding their capacity to solve the task has been reported. Here we investigated whether guppies (*Poecilia reticulata*) could discriminate stimuli based on their sameness in the sMTS where fish presented with a sample stimulus had to choose which of two simultaneously presented comparison stimuli matched it. We also assessed how performance was influenced by the training set size and stimulus type. Three experiments were conducted using three different sets of stimuli: two colors (red and green), two geometric shapes (circle vs. triangle); and multiple shapes. Performance was analyzed using binomial tests, t-tests, and generalized linear mixed models. The results showed that guppies learned to select the rewarding stimulus in a relatively limited number of trials and were successful in all experiments. Although no effect of the training set size was observed, guppies were more accurate when multiple stimuli were used. These findings support previous evidence suggesting that multiple training stimuli may improve generalization abilities and set the basis for future studies that adopt a delayed version of the task.

## Simple Summary

When it comes to making quick decisions based solely on a few visual features, fish have been shown in the literature to be, in general, skilled decision-makers. In this study, we investigated how fish are able to recognize two stimuli as identical while discarding a third one. We started by using basic features such as color and shape, and then assessed the same ability using a set of twelve different geometric figures. Although the fish were successful in all three tasks, they performed particularly well as the complexity increased, suggesting a strong ability to abstract essential visual features and use them to their advantage. Matching to sample is a tool that allows for a precise assessment of our ability to detect similarities and differences in a cognitive task. Identifying this ability in animal organisms helps us better understand the perceptual rules that guide visual behavior.

## 1. Introduction

Claims of human intellectual superiority have been rooted in the belief that a high level of cognitive processing is unique to humans. During the last century, comparative cognition studies offered valuable insights into the evolution and development of human cognition, showing similarities between humans and other animals’ cognitive abilities [1,2]. For instance, there is evidence of tool use in Caledonian crows and ravens [3], and metacognitive skills (the ability to monitor and regulate one’s internal cognitive processes) have been demonstrated in some nonhuman primates [4]. Although it is now widely accepted that mammals and birds possess complex cognitive capacities, fish have been largely overlooked. However, research on fish cognition is fundamental to understanding the evolutionary transitions of cognitive skills in vertebrates, as all tetrapods evolved from a common fish-like ancestor [5]. Furthermore, with over 30.000 species, fish represent half of the vertebrate species on the planet, are adapted to live in almost every aquatic habitat, and exhibit an extraordinary behavioral variety to cope with social and physical problems as complex as other vertebrates [6]. Hence, fish must be able to analyze, store, and recall information to survive and be optimally adapted to their environments. For these reasons, fish have become a model organism in comparative cognition research to investigate the role of phylogeny and ecology in the evolution of cognitive abilities [6].

Over the last few decades, a growing number of studies have demonstrated that fish possess a level of cognitive complexity previously underestimated, exhibiting abilities comparable to those of mammals and birds. For instance, there is evidence of numerical competence [7], mirror self-recognition [8], concept learning [9], and spatial learning [10]. These cognitive abilities likely evolved to help fish survive in their natural habitats, where they face daily challenges such as predation, mate choice, and competition for resources. Despite clear anatomical and morphological differences, the genetic, neuronal, and physiological mechanisms underlying fish behavior show strong similarities to those described in vertebrates [11]. Major forebrain subdivisions, such as the subpallium, the pallium, and the olfactory structures, are conserved in tetrapods. Recent comparative analyses of cell types and brain regions in the telencephalon across all five major vertebrate lineages revealed significant transcriptional similarities among them, providing new insight into the homologies in the vertebrate forebrain [12]. Furthermore, changes in visual numerosity that are encoded into the prefrontal and posterior parietal neocortex in humans and primates, selectively activate the caudal part of the dorso-central division of the zebrafish pallium, that corresponds topologically to the mammalian neocortex and is supposed to be functionally homologous [13]. These findings, together with evidence of the number of neurons in the caudal nidopallium (a higher associative area) of young domestic chicks [14] suggest that different brain structures functionally similar to the mammalian prefrontal cortex are involved in processing the same information in different animals [15,16,17]. 

Guppies (*Poecilia reticulata*) have largely contributed to increasing the current knowledge on fish cognition and behavior. Studies have shown that guppies exhibit various forms of learning: for instance, guppies can navigate complex mazes [18] and can learn from observing their conspecifics where to find food and how to avoid predators [19]. Several studies have reported problem-solving skills [20], and discrimination abilities when presented with stimuli differing in colors and shapes [21,22]. Guppies can also learn to discriminate between sets of abstract items differing in numerosities and generalize the learned rule (e.g., choose the larger set) to novel ones [23,24]. Although learning and generalization abilities in guppies have been largely demonstrated, no studies have investigated their ability to discriminate stimuli based on their sameness. 

Being able to sort objects into classes allows individuals to transfer learning to new stimuli perceived as similar to the old ones, thus sparing time and reducing costs related to new trial-and-error learning [25]. The ability to judge sameness relations requires assessing whether two objects are identical and then transferring this general rule to novel stimuli, thus showing that the learned concept is not bound to the specific stimuli in question [26,27]. A well-established paradigm used to investigate discriminative abilities based on the same/different concept is the simultaneous match-to-sample task (sMTS). In this task, subjects are first shown a stimulus (sample) and then can choose between two comparison stimuli presented simultaneously. The sample stimulus remains visible in the center of the two comparison stimuli at the time of choice. One of the comparison stimuli is identical to the sample (matching stimulus) whereas the other one is different (non-matching stimulus). Only the choice of the matching stimulus is rewarded. Although the ability to judge whether two things as different or the same has long been considered a hallmark of human cognition, evidence of successful performance in the sMTS task has been reported in different species including bees [28,29,30]. For instance, honeybees initially trained in an sMTS, where they had to match the comparison stimuli (blue and yellow) with the sample stimulus (either yellow or blue), were then tested with novel stimuli (vertical or horizontal black- and-white gratings). Honeybees chose the novel comparison stimulus (e.g., vertical gratings) that matched the sample, although they had no previous experience with the test stimuli. A similar pattern was observed in honeybees first trained in the sMTS using the grating stimuli and then tested with colors. These results showed that even animals with minimal neural architectures are capable of learning conceptual relationships.

Much attention has been devoted to understanding the mechanisms underlying same/different concept learning, particularly through studies on pigeons and primates. For instance, Wright and colleagues [28] reported that the set size used during training affected pigeons’ performance in the sMTS. In their study, a group of pigeons was trained with only two stimuli (apple and duck), and each stimulus appeared half the time as the sample and matching stimulus and half the time as the incorrect non-matching stimulus. A second group, instead, was trained with 152 different stimuli, and each stimulus appeared only once in each daily session. Once the learning criterion was reached, pigeons were tested in transfer trials with novel stimuli. Results showed that pigeons trained with only two alternating items learned the task faster (~1200 trials) compared to pigeons trained with a large set size of 152 items (~27.000 trials). However, only pigeons trained with the large set learned and employed the abstract concept of same/different when presented with novel stimuli, whereas the pigeons trained with the small set size, did not. These results showed that small training sets led to fast acquisition but little if any transfer to novel stimuli, whereas large sets led to slower learning but significant transfer to novel stimuli [31]. This was probably due to the fact that when a large set size was used, it was difficult for the subjects to focus on the absolute properties of the stimuli, remember them, and form item-specific associations, namely memorizing the position of each item in all displays that led to reinforcement. For instance, when only a few stimuli are used, only a limited number of configural patterns are possible. In this scenario, pigeons learn the unique pattern of each display and respond accordingly [32]. As a consequence, concept learning might have become an easier strategy to solve the task as the learned rule transcended the specific features of the training stimuli. Similar set-size effects when learning the same/different concept have been described in pigeons, nutcrackers, rhesus monkeys, magpies, and capuchin monkeys [27]. 

Cognitive abilities in fish have been extensively studied in different areas of research (e.g., social cognition, spatial learning, and numerical competence) using multiple experimental paradigms. However, to date, there is only a limited number of studies on sMTS in fish that have reported contrasting results. Evidence of successful performance in sMTS in goldfish was provided by Goldman and Shapiro [33] and Zerbolio and Royalty [34]. In the first study, fish trained using colored lights (red, green, and blue, alternatively used as samples) showed an accuracy of 75% at the end of the training (70 sessions, 120 trials per session) with some subjects performing above 85%. In the second study, goldfish trained with color lights (either blue/yellow or red/green) in an avoidance shuttle box, performed above chance level before the end of the training period (18 out of 30 days, 40 trials per day). More recently, zebrafish [35] also proved capable of passing the sMTS within 15 sessions (10 trials per session) using colored panels as stimuli (red and green). Malawi cichlids [36] instead did not learn to perform the sMTS when trained with two-dimensional shapes (a cross and a circle) for 40 sessions (10 trials per session). Similarly, archerfish [37] trained with three shapes (line drawings alternatively used as samples) were unable to learn the task within 40 sessions (10 trials per session). More recently, Aellen and colleagues [38] adopted a new approach and trained cleaner fish *Labroides dimidiatus* with a large number of different stimuli (400 shapes) using each stimulus only once as previous studies showed that multiple sample stimuli may enhance generalized rule learning [29,39]. They found that cleaner fish learned an abstract general rule and performed above chance in the sMTS in a relatively limited number of trials (200). However, as the authors did not compare the new procedure with the standard paradigm, with only two/three training stimuli, it was not possible to conclude that a large set size was more efficient in facilitating the acquisition of the task.

Animals continuously encounter same/different discriminations in nature (e.g., food selection, predator avoidance). For instance, if an individual finds a palatable food item, it is plausible that they would search for others that are similar. Hence, there is no reason to assume that the ability to discriminate stimuli based on sameness arose only in humans; rather, it is likely a mental toolkit shared across species. The results of the present study will not only contribute to increasing our knowledge of fish cognitive abilities but may also provide further insight into animals’ ability to distinguish sameness and difference to better understand the evolution of human and animal cognition.

Given the mixed results on fish, here, we investigated whether guppies could successfully perform in an sMTS and if performance varied depending on the training set-size and the type of stimuli presented. We designed a new protocol, and we conducted three experiments with different stimuli: in Experiment 1, one group of guppies was trained with two colors (red and green), in Experiment 2, another group was trained with two geometric shapes (circle vs. triangle) and in Experiment 3, a third group was trained with multiple shapes. If small set sizes speed up learning, we would expect the two-stimuli groups to learn faster compared to the multiple-stimuli group, whereas, on the contrary, subjects are expected to be more accurate as the larger number of stimuli would favor generalization skills.

## 2. Materials and Methods

### 2.1. Subjects

The study was conducted on adult female guppies, originating from an outbred domestic strain maintained at the Department of General Psychology, University of Padova. The guppies were acquired three months prior to the experiments from a local supplier and kept in a mixed-sex stock of approximately 100 adults. The fish were kept in a 400-L plastic tank (65 × 100 × 70 cm), enriched with aquatic plants (*Hygrophila corymbosa* and *Taxiphyllum barbieri*), a gravel substrate, and two biomechanical filters. Temperature was maintained at 26 ± 1 °C, and lighting followed a 12:12 h light/dark cycle using a 30 W phytostimulant lamp. Subjects were fed twice daily: commercial food flakes (Aqua Tropical, Padovan^®^) in the morning and live brine shrimp (*Artemia salina*) in the afternoon. A total of 55 experimentally naïve guppies were tested during the three experiments. Fish were randomly assigned to the three different experiments. For each experiment, testing proceeded until 10 subjects completed the training phase. Eight subjects failed to pass the habituation phase, and 17 could not complete the 20-day training phase as they stopped responding and were then reintroduced to the maintenance tanks.

The study aimed to investigate whether guppies could successfully perform in an sMTS task, a protocol commonly adopted with mammals and birds [30,40], to improve our knowledge about cognitive abilities in fish. For this reason, the species of interest could not be substituted by another one. Each apparatus was connected to a secondary aquarium housing conspecifics and to a filtration system, positioned below the experimental apparatuses, that provided social olfactory cues to avoid social isolation [41].

Experiments were conducted in agreement with the law of our country (Italy, D.L. 4 Marzo 2014, n. 26) and the Ethical Committee of the University of Padova reviewed and approved the experimental procedures. The subjects were not food deprived and were not forced to participate in the trials. At the end of the experiments, all subjects were released into the maintenance tanks.

### 2.2. Experimental Apparatus and Stimuli

**Figure 1:**
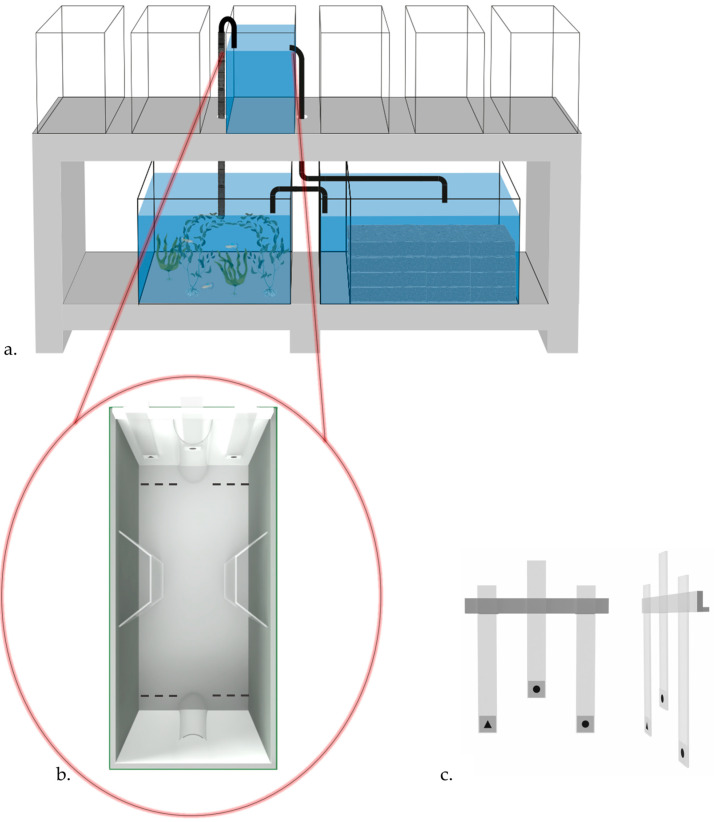
(**a**) Representation of the experimental set-up. (**b**) 3D representation of the experimental apparatus; two lateral compartments positioned halfway virtually divided the apparatus into three sections: a frontal, a posterior, and a central compartment. The dashed lines indicate the position of a transparent plastic strip that helps in determining the subject’s choice. (**c**) Front- and side-view of the three-panel structure used for stimulus presentation, showing a trial from Experiment 2 in which the circle represents the correct stimulus.

We adopted the experimental apparatus previously used to study visual discrimination abilities in guppies [42] consisting of glass tanks (20 × 50 × 32 cm) filled with 28 L of filtered water and enriched with loose gravel. Eighteen identical experimental tanks were used (six tanks per table). Each tank was kept in olfactory communication through a recirculating, silent filtering system connected to a large aquarium placed beneath the tanks that house a group of adult conspecifics (approx. 20 fish, Figure 1a).

Internally, each tank was shaped as an hourglass due to two lateral compartments (10 × 6 × 32 cm; Figure 1b) made with acetate sheets that were positioned in the center of the two long walls of the tank and that virtually divided the tank into three compartments: a frontal compartment, a posterior compartment, and a central compartment.

The tanks were surrounded with green plastic material to prevent the fish from being influenced by external visual stimuli, while internally two white plastic sheets were placed in order to enhance stimulus visibility. To prevent direct contact between the fish and the sample stimulus, a transparent semi-cylinder was placed on both short walls.

During the habituation phase, a single transparent panel (32.5 × 3.5 cm) was used. During the training trials, the stimuli were presented using a three-panel structure (Figure 1b) provided with a transparent pocket (3.5 × 3.5 cm) at the end of each panel to hold the stimulus. The central panel used to present the sample stimulus measured 25.5 × 3.5 cm, whereas the two lateral ones used for the comparison stimuli measured 32.5 × 3.5 cm.

**Figure 2:**
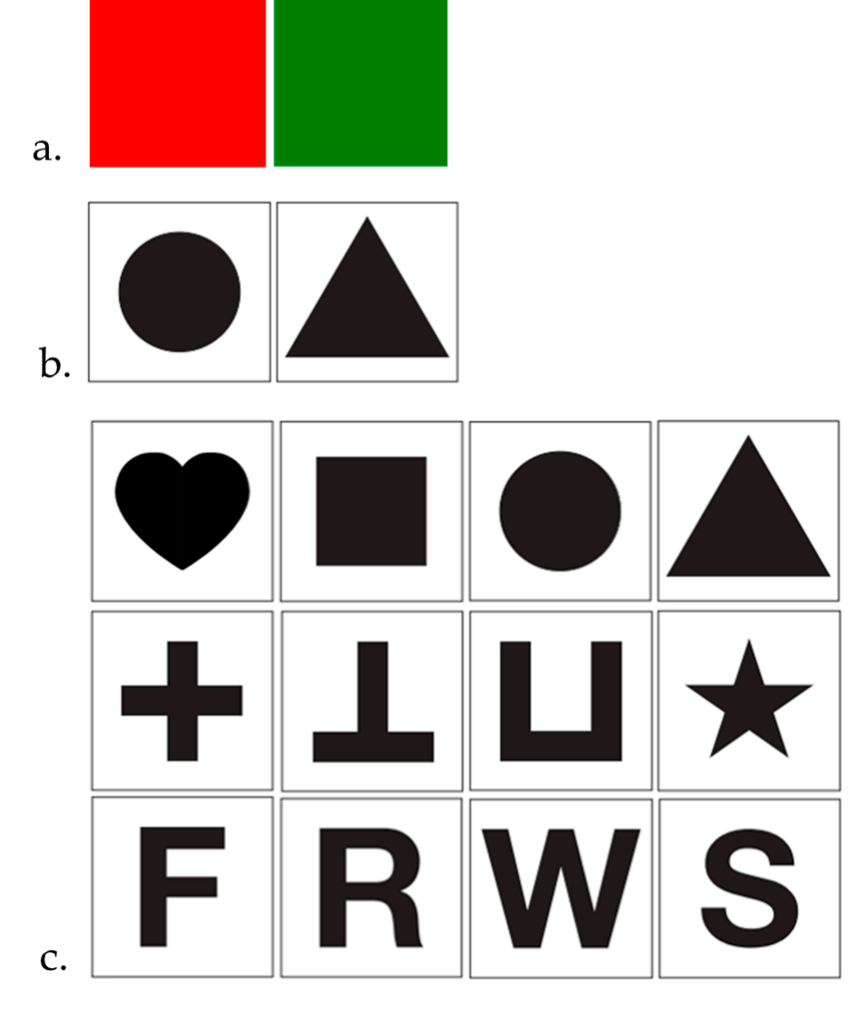
Experimental stimuli: (**a**) color-based matching (Exp. 1), (**b**) shape-based matching (Exp. 2), and (**c**) complex shapes discrimination (Exp. 3).

We derive a different set of stimuli starting from those previously adopted in *Malawi cichlids*, guppies, and zebrafish [36,43,44]. Stimuli were designed using Adobe Illustrator 26.0 and printed on laminated cards (3 × 3 cm). In Experiment 1, we presented red (RGB: 255, 0, 0) and green (RGB: 0, 128, 0) stimuli (Figure 2a). In Experiment 2, we used two black geometric shapes [43], a circle (⌀ 2 cm, area 3.142 cm^2^) and a triangle (2.3 × 2.7, area 3.157 cm^2^) with comparable areas to control for size-associated discrimination (Figure 2b). Finally, in Experiment 3, we presented a total of 12 complex shapes based on the set used by Gierszewski and colleagues [36], and we employed a plugin of Adobe Illustrator (GetShapeArea https://gist.github.com/bryanbuchanan/11387501, accessed on 10 May 2022) to calculate the area of each stimulus. This set of stimuli included both geometric figures (e.g., square and circle), and more abstract symbols (e.g., letters “F” and “S”, star, heart) (Figure 2c). The areas ranged from 1.376 to 3.24 cm^2^. The variety of stimuli used in this experiment was created to investigate the ability of subjects during a matching-to-sample task to differentiate between simple and complex visual shapes.

During the training trials, presentation of the matching (correct) and non-matching (incorrect) stimuli was balanced (left and right/front and back) and no more than twice in a row on the same side to avoid the development of a side bias.

Each daily training session consisted of 12 trials. In Experiment 1, red was presented as the sample stimulus in half of the trials, and green was the sample in the other half. In Experiment 2, the presentation of the sample was counterbalanced as in Experiment 1. In Experiment 3, each stimulus was used as a sample once per session so that all the stimuli were equally presented.

### 2.3. Procedure

**Figure 3:**
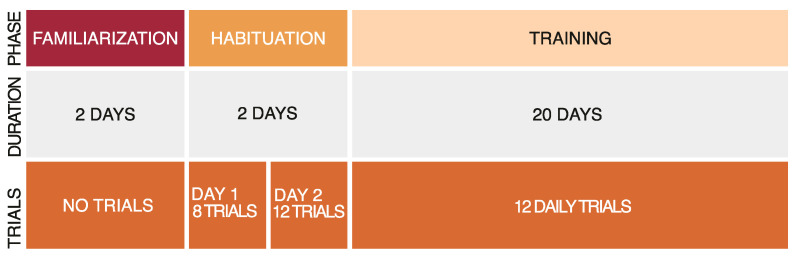
Schematic representation of the timeline for the three experiments.

We developed a novel sMTS procedure based on previous literature [42]. The procedure consisted of three different phases: familiarization phase, habituation, and training phase (Figure 3).

In the familiarization phase, subjects were individually moved to the experimental apparatus and allowed to acclimate for two days. During this period, they were fed once daily with *Artemia salina* nauplii, delivered into the central compartment to promote exploration and to avoid the development of side biases.

The habituation phase lasted two days. During the first day, subjects underwent eight trials divided into two 4-trial sessions separated by a 90-minute interval (inter-trial was 15 min). The second day, subjects underwent the same schedule, but the number of trials was twelve (six trials per session). During each trial, a transparent panel (32.5 × 3.5 cm) was alternatively placed on the right or left side of the tank, either on the frontal or on the posterior wall. Subjects had a maximum of 10 min to approach the panel; otherwise, it was removed, and the response was recorded as null. When approaching the panel, subjects received only a small amount of food reward to avoid satiation (a drop of *Artemia salina* nauplii was released with a 3 mL Pasteur pipette). The panel remained in place until the reward was consumed (approximately 3–5 min). At the end of this second phase, we excluded those subjects that did not approach the panel in at least 10 out of 12 trials on the second (and final) day of this phase.

The training phase consisted of 12 daily trials for a total of 20 days. Training sessions took place from Monday through Saturday, within the time window 9:00 to 16:30. Trials started when the three-panel structure was placed inside the tank, simultaneously showing the sample stimulus and the two lateral stimuli. The structure was positioned so that the sample was inserted in the transparent semicylinder to prevent the subjects from approaching it. Subjects had a maximum of 5 min to make a choice (defined as being within one body length close to the matching or non-matching stimulus, see Figure 1b). If no choice was made within this interval, the trial was recorded as null, and the stimuli were removed. A correct choice was food-rewarded by releasing a small amount of *A. salina* close to the correct stimulus. Stimuli were kept in place until the subject consumed the reward. After an incorrect choice, the three-panel structure was immediately removed.

### 2.4. Data Collection and Statistical Analysis

During each trial, subjects’ behavior was recorded manually. The choice was determined as the subject being at least one body length from one of the two lateral stimuli (see Figure 1b). Trials in which subjects did not make a choice were recorded as null (NA) and excluded from the analysis. When subjects chose the matching stimulus, the response was recorded as correct (1), whereas choosing the non-matching stimulus was recorded as incorrect (0). Hence, the subjects’ performance was expressed in a binary metric. In Experiment 2, two subjects did not make a choice in 8 and 17 trials, leading to the exclusion of 25 out of 2400 trials from the dataset.

We analyzed both the overall group performance and the individual performance in each experiment, using RStudio, version 4.4.2 (R Foundation for Statistical Computing, Vienna, Austria).

Group-level analyses were conducted to assess whether overall subjects learnt to perform the sMTS task both at the end of the 20-day training period and before the conclusion of the experiment. Hence, we calculated the mean proportion of correct choices of each subject: for the entire duration of the training and for blocks of five days (block 1: days 1–5; block 2: days 6–10; block 3: days 11–15; and block 4: days 16–20). Therefore, we first conducted a Shapiro–Wilk test to determine if the data were normally distributed. Secondly, to determine if there was a difference from chance level (0.5), we performed a two-tailed one-sample *t*-test. In Experiment 3, block 1 was not normally distributed; therefore, we performed the Wilcoxon-Mann-Whitney test. Significance threshold was *p* = 0.05.

Individual analyses were performed using a binomial test. For each subject, we counted the total number of both correct and incorrect choices during the entire period of training, and we tested whether the number of correct trials was significantly above the chance level (0.5).

To further investigate performance, a generalized linear mixed-effects model (GLMM) was fitted for each experiment using the glmer function from the lme4 package with a binomial family and logit link function. The models included subjects’ identification code as a random effect, and blocks of 5 days and stimulus type as fixed effects, to investigate if these variables influenced the performance. When the stimulus was significant, post hoc comparison was conducted using the emmeans function, and Tukey’s adjustment was used to adjust for multiple comparisons.

Lastly, to analyze data across all three experiments together, we fitted a GLMM including subjects’ ID nested within the experiment as random effects, and experiment and blocks of 5 days as fixed effects.

## 3. Results

### 3.1. Experiment 1

**Figure 4:**
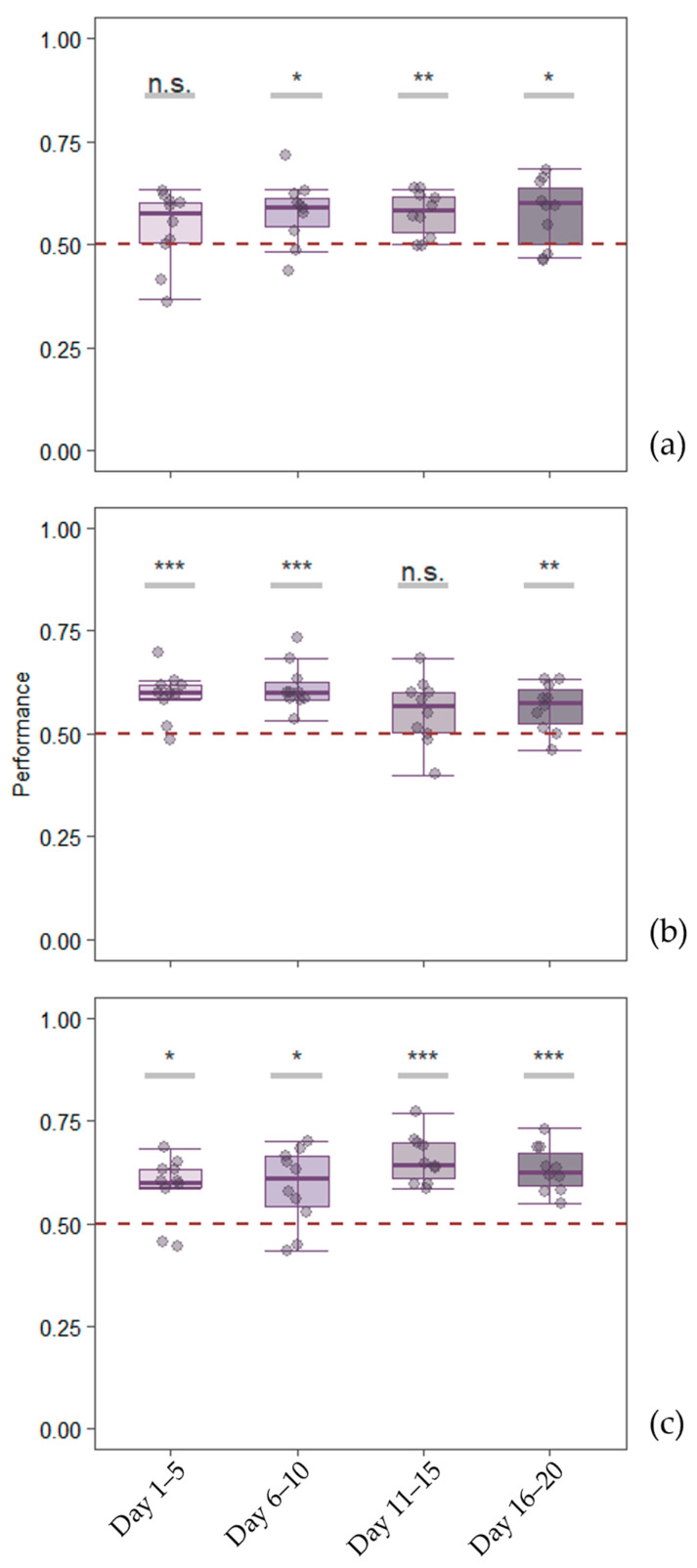
Boxplots showing group performance across the four 5-day blocks for (**a**) Experiment 1, (**b**) Experiment 2, and (**c**) Experiment 3. Individual data points are represented by dots. The median (solid line) and the chance level (0.5, dashed line) are reported. Asterisks indicate statistical significance (n.s. = not significant, * *p* < 0.05, ** *p* < 0.01, *** *p* < 0.001).

Group level analyses showed that overall, subjects were able to perform the sMTS task with red and green stimuli (0.57 ± 0.042 of correct choices; t_9_ = 5.1355, *p* < 0.001). When we analyzed data separately for blocks, subjects successfully choose more frequently the matching stimulus in all blocks (t_9_ = 3.121, *p* = 0.012; t_9_ = 4.4388, *p* = 0.002 and t_9_ = 2.9665, *p* = 0.016 for block 2, 3 and 4 respectively, see Figure 4a) but block 1 (t_9_ = 1.4056, *p* = 0.193).

Individual analyses showed that the performance of 7 out of 10 subjects was significantly above chance level (see Table 1 for details).

Results from the GLMM revealed that stimulus type had a significant effect (χ^2^_1_ = 5.7073, *p* = 0.017), and post-hoc comparisons showed that performance was significantly higher when subjects had to match the green stimulus (estimate = −0.199, SE = 0.0827, *p* = 0.016). No significant effect of blocks (χ^2^_1_ = 1.3913, *p* = 0.238) nor the interaction between stimulus type and blocks emerged (χ^2^_1_ = 1.8113, *p* = 0.178).

### 3.2. Experiment 2

Group-level analyses showed that subjects succeeded in the task using geometric shapes (0.58 ± 0.042; t_9_ = 6.1664, *p* < 0.001). When considering the blocks, a significant performance was observed in block 1, 2 and 4 (t_9_ = 5.0203, *p* < 0.001; t_9_ = 6.2777, *p* < 0.001 and t_9_ = 3.5221, *p* = 0.006 respectively) but not in block 3 (t_9_ = 2.087, *p* = 0.067; Figure 4b).

Individual performance analysis showed that 5 out of 10 subjects performed significantly better than chance level (see Table 1).

The GLMM revealed no significant effect of blocks (χ^2^_1_ = 2.4988, *p* = 0.114) nor the interaction between blocks and stimulus (χ^2^_1_ = 3.6263, *p* = 0.057). There is a significant effect of the stimulus type (χ^2^_1_ = 16.0129, *p* < 0.001), with subjects performing better when subjects had to match the circle (estimate = 0.334, SE = 0.0837, *p* < 0.001).

### 3.3. Experiment 3

In Experiment 3, group-level analyses revealed that subjects were able to perform the task using complex shapes (0.62 ± 0.048; t_9_ = 7.6828, *p* < 0.001). Considering the four 5-day blocks, subjects’ performance was consistently above chance level (V = 52, *p* = 0.014, t_9_ = 3.0166, *p* = 0.015, t_9_ = 8.5654, *p* < 0.001 and t_9_ = 7.5362, *p* < 0.001 for blocks 1, 2, 3 and 4 respectively, Figure 4c).

At the individual level, 8 out of 10 subjects performed significantly above chance level (see Table 1).

The model showed no significant effects of stimulus and the interaction between stimulus and blocks (χ^2^_11_ = 12.9274, *p* = 0.298; χ^2^_11_ = 10.4792, *p* = 0.488, respectively). However, there was a significant effect of the main factor ‘blocks’ (χ^2^_1_ = 4.6914, *p* = 0.03), indicating an improvement during the training period.

### 3.4. Comparison Among Experiments

**Figure 5:**
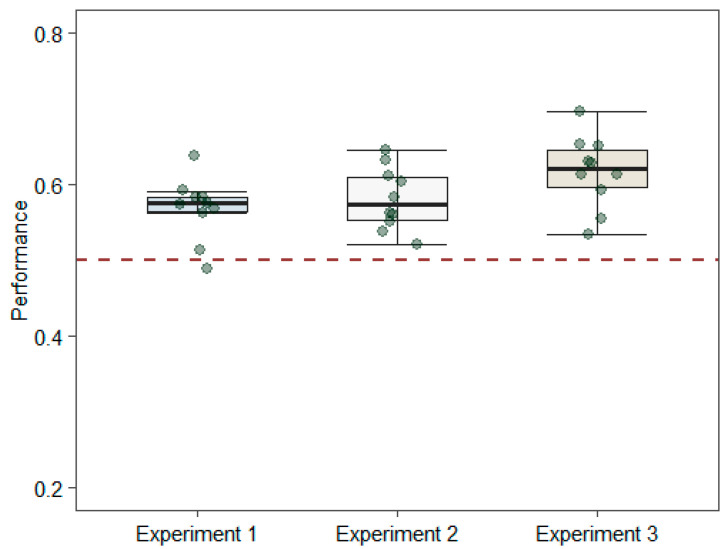
Boxplots showing the mean performance of subjects for each experiment. Dots represent mean individual performance. The solid line within each boxplot indicates the median performance, and the red dashed line represents the chance level.

Considering all the experiments together, the model showed no significant effect of the main factor ‘blocks’ (χ^2^_1_ = 1.0733, *p* = 0.3). However, a significant effect was seen on the interaction between blocks and experiment (χ^2^_2_ = 7.6645, *p* = 0.022) and the main factor of experiment (χ^2^_2_ = 7.2915, *p* = 0.026; Figure 5). Subjects’ performance on Experiment 1 (color stimuli) was significantly lower than subjects’ performance on Experiment 3 (complex shapes) (estimate = −0.2037, SE = 0.0773, *p* = 0.023). No differences were found concerning the other contrasts (all *p* values > 0.05).

## 4. Discussion

In this study, we evaluated the ability of guppies to perform a discrimination task using the sMTS and investigated whether performance could vary based on the nature of the stimuli and the training set size. Visual discrimination has been extensively studied in guppies using a standard approach that consisted of the presentation of two stimuli, and fish had to learn that within each pair of stimuli, one was rewarding whereas the other was not. There is compelling evidence that this species is capable of discriminating stimuli varying in colors, numerosity, shapes, and complexity using this approach, showing surprising cognitive abilities and challenging long-held beliefs about fish cognition [43,45]. Here we showed for the first time that guppies can also learn to discriminate whether two stimuli were the same or different based on their physical properties in the sMTS task, a completely novel paradigm for this species, where they had to match three stimuli (one sample and two comparing stimuli) to make their choices. In fact, overall, guppies successfully learned the task in all the experiments although with some differences in performance. 

When colors were used as stimuli in Experiment 1, fish significantly discriminated between green and red starting from the second block of training showing that learning occurred more slowly in comparison with Experiment 2, where the same set-size stimuli were used but performance was above chance level already during the first block. Recently, Gatto and colleagues [45], demonstrated that guppies achieved the red/green discrimination in a relatively large number of trials (on average, 416 ± 266.64) compared to shape discrimination (circle vs. triangle: on average, 225.60 ± 156.19) using an automated training device where stimuli were presented on a screen. Despite there is evidence that differences in experimental design may cause significant differences in performance [46,47,48], our results aligned with the previous findings, suggesting that discrimination between red and green might be compelling for guppies. With respect to the selected colors, it has been shown that this species can quickly learn to discriminate red and yellow [21,43]. This is probably due to the salience of these colors in their natural habitats as guppies have a natural preference for small carotenoid-rich fruits that drop into the rivers where they live [49]. Anyway, as red/yellow discrimination was achieved in a very few trials, we decided to select other colors in accordance with previous literature to make the shape and color discrimination more comparable in terms of learning rate. 

For what concerns shape discrimination, guppies quickly learned the task both when presented with 2 stimuli in Experiment 2 and when multiple stimuli were used in Experiment 3, despite previous findings showing that the training set-size could affect the acquisition of the task [28]. A possible explanation may be related to the remarkable capacity of this species to recognize individual conspecifics relying on visual cues. Studies have reported that guppies have a sophisticated memory, being able to recognize dozens of conspecifics over extended periods [50,51], a capacity that could play a crucial role in various social contexts, including mate choice and kin recognition [52]. Hence, we cannot exclude that the sample size used was not perceived as more complex to memorize than the 2-shape set, and that guppies learned to solve the task by memorizing each pattern to make the correct decision, as reported in pigeons [53]. Future studies with a larger number of stimuli are necessary to fully compare the effect of the training set-size on learning in this species. Nevertheless, although not significantly, subjects were more accurate in Experiment 3 and improved their performance over trials contrary to Experiment 2. The lack of improvement in Experiment 2, may be due to reduced motivation resulting from the constant use of the same stimuli, whereas the presentation of multiple items might have required long-term attention and favored generalization skills in order to obtain a food reward in a more cognitively demanding context. 

A further aspect to take into consideration is that interindividual variability emerged in the three experiments. In fact, a slightly larger number of fish successfully performed the sMTS task in Experiments 1 and 3 compared to Experiment 2 (7 out of 10 in Experiment 1, 5 out of 10 in Experiment 2, and 8 out of 10 in Experiment 3). This should not be surprising as interindividual differences have been widely described in cognitive studies both in humans and non-human animals [54,55,56,57] leading researchers to argue that interindividual variability not only influences immediate behavior but also has broader implications for the species’ evolution and adaptation [58,59]. Within this context, several studies have shown that guppies also exhibit consistent individual differences in different tasks, such as spatial learning, long-term memory retention, and cognitive flexibility [60,61]. Interestingly, a recent study investigated the relationship between variation in brain morphology and cognitive abilities to understand inter-individual variation in guppies’ cognition [62]. Guppies were first tested in a color-discrimination learning task to assess their associative learning ability and then underwent the reversal learning task to evaluate their cognitive flexibility. Individual-level analyses showed that optic tectum relative size and relative telencephalon size, correlated positively with discrimination learning and reversal learning, respectively. These results provide evidence that inter-individual variability in brain region size underlies individual variation in cognitive abilities. A subsequent study confirmed that individual differences in telencephalon relative size underpin inter-individual variability in executive function capacities. Guppies with relatively larger telencephalons performed better than those with smaller telencephalons in three assays measuring different executive functions: cognitive flexibility, inhibitory control, and working memory [63]. It would be interesting to perform the sMTS task with up- and down- artificially selected lines for the telencephalon size to test the possibility that the variation in brain morphology may explain the variability observed in the present study. It is now widely acknowledged that even if a few individuals within a species can master a particular task, it represents a significant indicator of the species’ cognitive potential as it implies that the underlying neural architecture necessary to support such ability exists within that species [64,65,66,67]. Hence, despite the variation in performance we observed, this study demonstrates that this species has the capacity to perform the sMTS contrary to other fish species [36,37].

However, we cannot claim that guppies learned the same/different concept. Different strategies can be adopted to solve the sMTS. One, as previously mentioned, consists of learning the unique pattern of each display and responding accordingly. Another way is to compare the stimulus features (e.g., the color of the shape) and learn a sample-specific if-then rule that guides following decisions (“if the sample stimulus is green, then choose comparison green”, “if the sample is circle, then choose comparison circle”). Finally, the task could be solved through relational concept learning in which one stimulus is categorized relative to another one irrespective of their individual features. Hence, the subject judges the relationships among stimuli that transcend stimulus specifics. The first two strategies are linked to each set of stimuli whereas a learned concept is a rule for accurately dealing with new stimuli without the need for retraining [68]. A fundamental criterion to ensure that animals achieve abstract concept learning is that subjects must undergo a transfer test, and they should perform as accurately with completely novel stimuli as with the training ones [27,30,68]. Here, guppies were not tested in a transfer task, as cleaner fish were not in the study by Aellen and colleagues [38], and their performance could be explained in terms of choices based upon common perceptual features between the stimuli. However, our aim was to investigate if they could perform a visual discrimination task using the sMTS, a paradigm that provided mixed results in previous fish studies and that was completely novel for guppies. In fact, here fish are required to compare three stimuli in order to make a choice, while the standard approach used to study their visual discrimination abilities typically involves presenting a pair of stimuli where only one is correct and is rewarded. Our results showed that, overall, guppies learned the sMTS in a relatively limited number of trials paving the way for future studies with the new protocol to investigate proper abstract-concept learning by including transfer trials with completely novel stimuli [27]. Moreover, only female guppies were tested in this study. While this choice was made to reduce variability and is common in training studies with this species [45,48], it may limit the generalizability of the findings in terms of their respective visual learning ability. Future research can be conducted to explore whether similar patterns emerge in males. 

Short-term memory can be studied using the delayed MTS (dMTS), where, contrary to the sMTS, there is a delay between the offset of the sample and the onset of the comparison stimuli. Hence, only the comparison stimuli are visible at the time of choice, and the subject has to remember the sample stimulus in order to choose the matching one [69,70]. Despite being used in several species [69], to date, only one study showed that zebrafish could execute the dMTS with a delay of 3–4 s using colors as stimuli (red vs. green) [35]. The finding that guppies can learn to discriminate both colors and shapes in the sMTS makes it possible to extend the range of stimuli used in the delayed version of the paradigm to investigate short-term memory in this species. 

A prime concern of comparative cognition is to pinpoint differences or similarities between human and animal cognitive abilities to find out how cognition has evolved. The field over the last few decades has focused particularly on studying cognition beyond humans due to the increased awareness that animals deserve consideration on their own. Consequently, we have witnessed a broadening in the phylogenetic map of animal cognition studies, aimed at understanding both species’ distinctive cognitive abilities and shared skills across species. Despite the efforts, most research remains concentrated within a few species with a strong bias toward studying birds and mammals. Ectothermic animals, in particular reptiles and amphibians, have been poorly investigated, limiting our ability to identify fundamental principles underlying vertebrate cognition. Recent studies have shown that these animals possess impressive cognitive skills, including problem-solving abilities, quantity discrimination, and social learning [71,72,73,74].

Yet, research on the ability to perform the matching to sample is missing in reptiles and amphibians. Inclusion of these taxonomic groups in this area of research is essential to enrich our understanding of the role of phylogeny and ecology in the evolution of animal cognition.

## 5. Conclusions

Our findings indicate that guppies can successfully perform the sMTS and learn to select the rewarding stimulus in a relatively limited number of trials. Furthermore, performance was consistent across the tasks, although accuracy was higher when multiple stimuli were used, suggesting increased generalization abilities with a larger training set size. These results highlight the potential of guppies as a valuable model for investigating more complex cognitive processes and their underlying mechanisms. The limitations of this study also suggest directions for future research. While this study focused on the sMTS task, adding a delay between the comparison of the sample and the presentation of the comparing stimuli, it has been proven to be a reliable tool to investigate short-term memory also in this species. Moreover, introducing a transfer test would allow researchers to assess whether guppies can learn the abstract concept of same/different. Finally, since we tested only females to minimize variability, future research could explore whether similar performance patterns are observed in males. Overall, this work provides a solid foundation for future investigations into the cognitive abilities of guppies.

## Figures and Tables

**Table 1:** Individual performance across the three experiments. The table reports the mean percentage of correct choices for each subject across the 20-day training period and the corresponding *p*-value from the binomial test.

Experiment	Subject	% Correct Choice	*p*.Value
Experiment 1	1	57.5%	**0.024**
	2	51.3%	0.747
	3	56.7%	**0.045**
	4	63.8%	**<0.001**
	5	56.3%	0.061
	6	48.8%	0.747
	7	59.2%	**0.005**
	8	58.3%	**0.012**
	9	58.3%	**0.012**
	10	57.5%	**0.024**
Experiment 2	1	53.9%	0.264
	2	56.1%	0.081
	3	60.4%	**0.002**
	4	64.6%	**<0.001**
	5	55%	0.137
	6	58.3%	**0.012**
	7	56.3%	0.061
	8	52.1%	0.561
	9	63.3%	**<0.001**
	10	61.3%	**0.001**
Experiment 3	1	65.4%	**<0.001**
	2	62.9%	**<0.001**
	3	61.3%	**0.001**
	4	61.3%	**0.001**
	5	53.3%	0.333
	6	59.2%	**0.005**
	7	62.9%	**<0.001**
	8	65%	**<0.001**
	9	55.4%	0.106
	10	69.6%	**<0.001**

## Data Availability

The raw data supporting the conclusions of this article will be made available by the authors on request.
